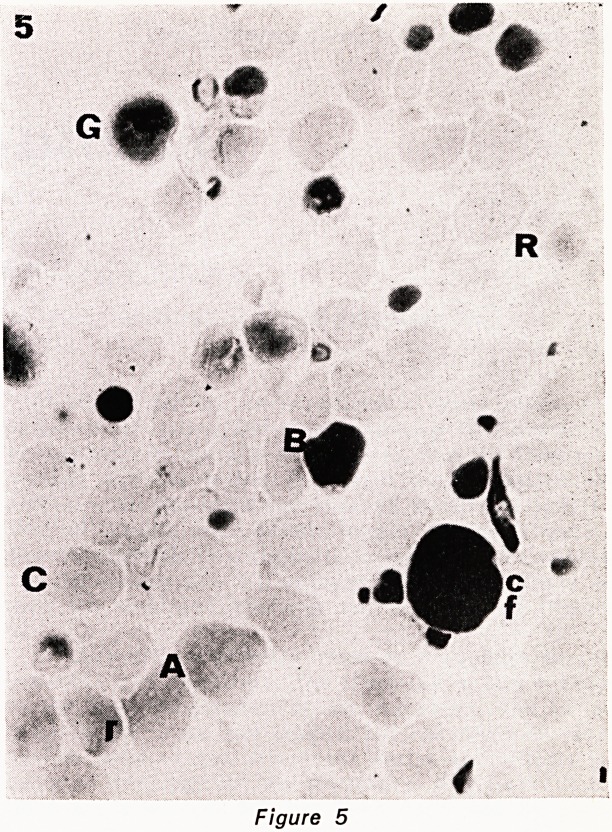# Histochemical Fibre Types in Human Extraocular Muscle

**Published:** 1975-04

**Authors:** D. G. F. Harriman

**Affiliations:** Neuropathology Laboratory, The School of Medicine, Leeds 2


### Histochemical Fibre Types in Human Extraocular Muscle

By

**D. G. F. Harriman, M.D., F.R.C.P., F.R.C.Path.**

Neuropathology Laboratory, The School of Medicine, Leeds 2.

It has long been known that the striated muscle fibres of which the extraocular muscles are composed differ in many respects from skeletal muscle in general. Their relatively small size, rich innervation, their small motor units and the presence of a proportion of fibres with multiple endplates are special characteristics which are recounted in most modern texts of ophthalmology. There are three sizes of extraocular muscle fibre, but pathologists called upon to examine biopsies of extraocular muscle are sometimes unaware of the normal fibre calibre variation and are tempted to make a diagnosis of neurogenic atrophy because of a supposed grouping of fibres of small calibre, or of myopathy when they see fibres of widely differing calibre and ring fibres. It is a truism to repeat that the normal must be known and recognised before pathology can be described.

In recent years the application of histochemical techniques to skeletal muscle has shown that the uniformity demonstrated by routine stains conceals two populations of muscle fibres of differing structure. Stains for oxidative enzymes and for myosin ATPase reveal two main types of fibre, randomly mixed in human muscles, and likely to correspond to the "red" and "white" muscle of many mammals. The mitochondria-rich fibres containing acid-stable myosin ATPase are variously referred to as type-1 or B fibres, and the mitochondria-poor, phosphorylase-rich fibres containing alkali-stable myosin ATPase are type-2 or A fibres. In many mammals intermediate or C forms exist, but these are not identical with the variations of type-2 fibres which have also been described (Brooke and Kaiser, 1970). There is general agreement concerning the two main types of muscle fibre, but not about their subdivisions.

When histochemical techniques are applied to extraocular muscle the situation is more complex still, and there is more variation between species. Miller (1967) described cell types with "varying mitochondrial configuration and histochemical characteristics" in rhesus extraocular muscle, and Asmussen *et al* (1971) in cat, rabbit, guinea-pig and rat concluded that there were at least 6 different fibre types. The full battery of histochemical reactions now considered necessary for fibre typing was not available to these authors, and both were concerned to draw conclusions from structure to function, assessing some fibres as slow, like those of frog muscle, and others as normal twitch fibres (slow and fast). Experience teaches that deductions regarding function from structure are liable to error (Huxley, 1962), and the functional characteristics of a given type of muscle fibre have to be determined by direct experiment. Both papers did establish however that the fibre composition varies in different parts of extraocular muscle, there being a peripheral zone generally composed of smaller fibres and a central area or core of fibres of varying size including large ones. Again there are species differences, rhesus muscle showing an additional intermediate zone. The complex findings apply to the rectus and oblique muscles, but not to the levator which is of simpler composition more like limb skeletal muscle.

The clearest exposition of fibre typing in extraocular muscle and one that employed a full battery of stains was that of Harker (1972). He found in sheep that there was a central core of mainly larger fibres, a peripheral (orbital rim) region of smaller fibres and an additional peripheral patch layer at the origin and insertion ends of the muscle, outside the orbital rim. Using differences in fibre diameter and histochemical profile he described six major fibre types, four of which were present in the central core. The largest fibres (7%) he termed G fibres, as these were shown to receive multiple small grape-like endplates like that of amphibian slow muscle. They showed little activity with phosphorylase, with oxidative enzymes (succinate dehydrogenase) or alkali-stable ATPase, but reacted strongly with acid ATPase. There were also large type A fibres (45%), having high activity with phosphorylase and alkali-stable ATPase and low activity with SDH and acid ATPase, and medium and small-sized type C fibres (48%). These had intermediate or high activity with phosphorylase, SDH and alkaline ATPase and a low reaction with acid ATPase. In the orbital rim there were equal numbers of medium C fibres, small C fibres and small G fibres.

Our findings in human extraocular muscle differ from those in the sheep. They are based on a single specimen of fresh inferior oblique muscle, provided by Mr. Brian Harcourt FRCS, following enucleation of the eye for melanoma in a man aged 30 years. Trials with post-mortem specimens were not found satisfactory. The entire muscle was quenched in isopentane cooled by liquid nitrogen, and serial $10\mu$ transverse sections of the muscle belly were cut in a Dittes cryostat. Successive sections were stained by HE, for phosphorylase, for NADH, for ATPase with preincubation at pH 9.4, 4.3 and 4.65, for lipids, and by the PAS and Gomori trichrome methods.

As in other mammalian species the muscle belly showed two major zones, an outer rim of smaller fibres and a large central core of fibres of more variable size. The zones were less well defined than in the sheep, the outer forming a band having a width about one-quarter of the lesser diameter of the muscle. The peripheral zone muscle fibres were closely packed and the central dispersed, accompanied by more connective tissue. There were several striking differences between the extraocular muscle and normal adult human limb skeletal muscle stained in the same way. Extraocular muscle fibres were in general much smaller, only the largest being as large as the smallest limb fibres. Roughly one-third of the fibres showed prominent staining of a thick intermyofibrillar network, visible even in HE sections (fig. 1). This is not seen in normal limb muscle. The myofibrils of these fibres formed thick masses in myofibrillar stains (fig. 4) and the intermyofibrillar network and mitochondria were prominent (fig. 3), in contrast to the fine stippling pattern of normal type-B (type-1) fibres. The coarse myofibril fibres resemble the *feldenstruktur* slow fibres of amphibian muscle, and have been claimed to receive multiple grape-like motor endings (Dietert, 1965). As already emphasised, their physiological properties remain an open question in human muscle.

A third unusual feature was the presence throughout the muscle of a small number of ring fibres. These are malformed muscle cells in which peripheral myofibrils spiral around a central bunch of normally orientated fibrils, and are found in normal limb muscle only near myotendinious insertions. They increase in number with age in human extraocular muscle. (Fig. 1-5).

Six types of muscle fibre were defined in the histochemical series. In the central zone 27% were type-A fibres, almost all of medium size, and 15% were of type-B (low phosphorylase and alkaline ATPase, high activity with NADH and acid ATPase). Type-C or intermediate fibres were in the majority (56%) and of these over half had a coarse *feldenstruktur* pattern. Thus *feldenstruktur* fibres were confined to type-C. The final variety of fibre seen in the central zone was the G fibre, reacting strongly with acid ATPase only. There were only 2 in 100 fibres, one of medium size and the other small.

In the peripheral zone there were 4% type-A fibres, 19% type-B, 74% type-C of which one-quarter were *feldenstruktur* fibres, and 3% small G.

Thus the human specimen examined included muscle fibres of the two main types common to limb skeletal muscle, and four types not found there, namely C fibres, *feldenstruktur* C fibres, medium and small G fibres. It remains to be seen whether this pattern is repeated in future examinations. The unique extraocular fibres exhibiting a strong reaction for both acid and alkaline ATPase noted in rat eye muscle (Yellin, 1969) are yet to be found in human muscle.

Figure 1

Figure 2

*Figure 3*

*Figure 4*

*Figure 5*

**Figures 1—5. Serial cryostat sections of central part of inferior oblique muscle, stained as follows: 1. HE. 2. phosphorylase. 3. NADH. 4. ATPase pH 9.4. 5. ATPase pH 4.3. The various fibre types are indicated by a letter to their immediate *left*. A= type A fibre. B=type B fibre. C=type C fibre. cf=type C feldenstruktur fibre. G=large G (grape-ending) fibre. g=small G fibre. R=ring fibre. All x 450.**

**REFERENCES**

ASMUSSEN, G. Kiesling, A. and Wohlnab, F., Histochemische Charakterisierung der verschiedenen Muskelfasertypen in den äuszeren Augenmuskem von säugetieren. Acta anatomica **79**: 526-545 (1971).

BROOKE, M. H. and Kaiser, K. K. Muscle fibre types: how many and what kind? Archs. Neurol. (Chic.) **23**: 369-379 (1970).

DIETERT, S. E. The demonstration of different types of muscle fibres in human extraocular muscle by electron microscopy and cholinesterase staining. Invest. Ophthal. **4**: 51-63 (1965).

HARKER, D. W. The structure and innervation of sheep superior rectus and levator palpebrae extraocular muscles. 1. Extrafusal muscle fibres. Invest. Ophthal. **11**: 956-969 (1972).

HUXLEY, A. F. Skeletal muscle. in Rodahl, K. and Horvath, S. M., editors: **MUSCLE AS A TISSUE**. New York, McGraw Hill. p.9 (1962).

MILLER, J. E. Scellular organisation of rhesus extraocular muscle. Invest. Ophthal. **6**: 18-39 (1967).

YELLIN, H. Unique intrafusal and extraocular muscle fibres exhibiting dual actomyosin ATPase activity. Exper. Neurol. **25**: 153-163 (1969).

## Figures and Tables

**Figure 1 f1:**
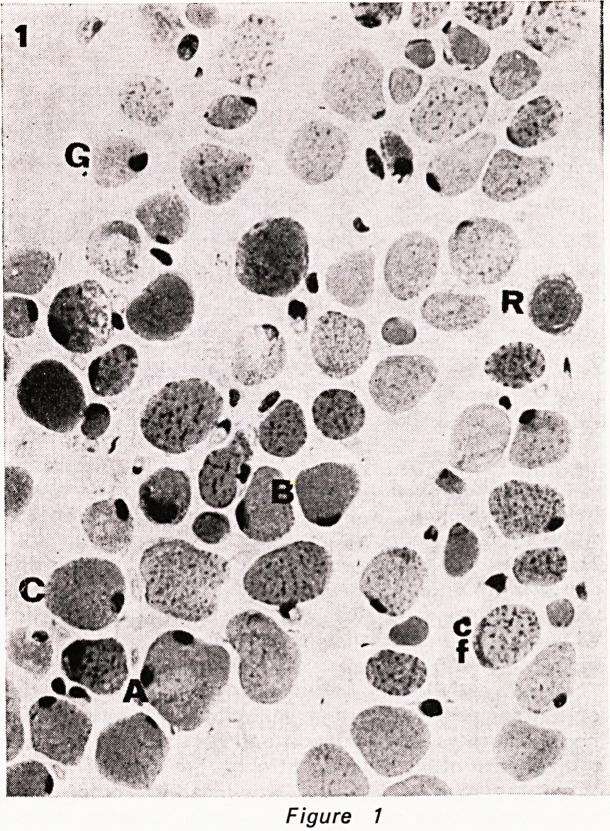


**Figure 2 f2:**
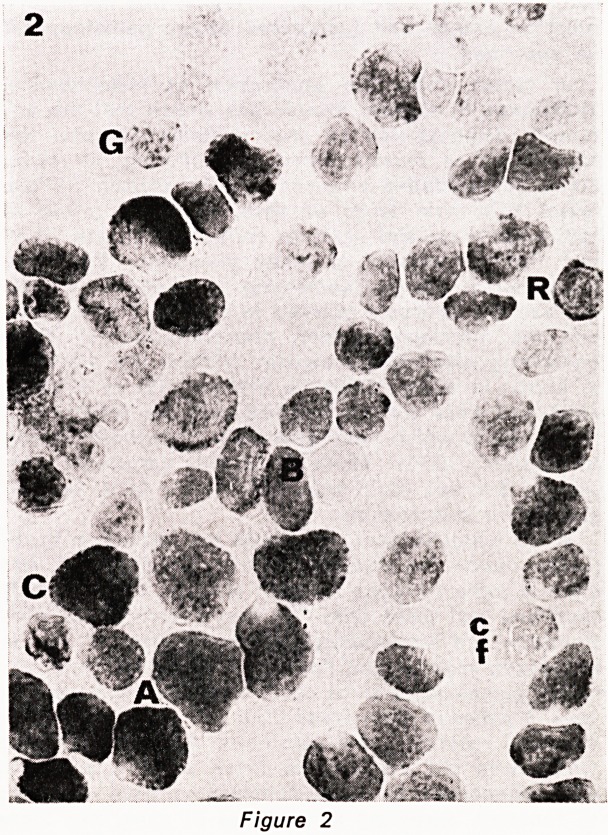


**Figure 3 f3:**
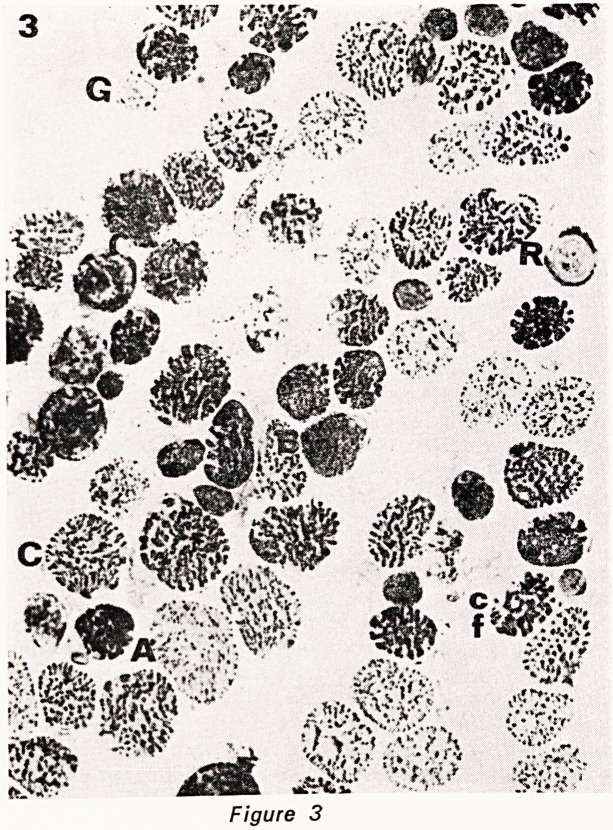


**Figure 4 f4:**
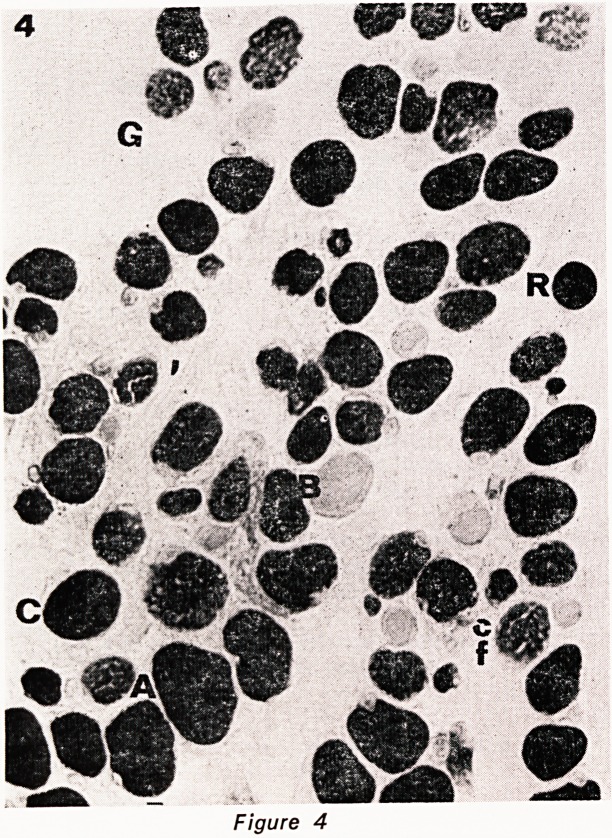


**Figure 5 f5:**